# The Impact of BKI-1294 Therapy in Mice Infected With the Apicomplexan Parasite *Neospora caninum* and Re-infected During Pregnancy

**DOI:** 10.3389/fvets.2020.587570

**Published:** 2020-10-15

**Authors:** Pablo Winzer, Dennis Imhof, Nicoleta Anghel, Dominic Ritler, Joachim Müller, Ghalia Boubaker, Adriana Aguado-Martinez, Luis-Miguel Ortega-Mora, Kayode K. Ojo, Wesley C. VanVoorhis, Andrew Hemphill

**Affiliations:** ^1^Institute of Parasitology, Vetsuisse Faculty, University of Bern, Bern, Switzerland; ^2^Graduate School for Cellular and Biomedical Sciences, University of Bern, Bern, Switzerland; ^3^Saluvet, Animal Health Department, Faculty of Veterinary Sciences, Complutense University of Madrid, Ciudad Universitaria s/n, Madrid, Spain; ^4^Center for Emerging and Re-emerging Infectious Diseases (CERID), Division of Allergy and Infectious Diseases, Department of Medicine, University of Washington, Seattle, WA, United States; ^5^Departments of Global Health and Microbiology, University of Washington, Seattle, WA, United States

**Keywords:** neosporosis, mouse model, bumped kinase inhibitor, calcium-dependent protein kinase, immune response, live-vaccine, real time PCR, proteomics

## Abstract

Exposure of *Neospora caninum* tachyzoites to BKI-1294 *in vitro* results in the formation of long-lived multinucleated complexes (MNCs). However, *in vivo* treatment of BALB/c mice with BKI-1294 shortly after *N. caninum* infection during pregnancy was safe and profoundly reduced pup mortality and vertical transmission. We hypothesized that the formation of MNCs could trigger immune responses that contribute to BKI efficacy *in vivo*. In this study, mice were first vaccinated with a sublethal dose of *N. caninum* tachyzoites and were treated with BKI-1294. We then investigated the effects of these treatments after mating and re-infection during pregnancy. Effects on fertility, pup survival, vertical transmission, and parasite load in dams were evaluated. Cytokines in sera or splenocyte culture supernatants were assessed by either ELISA or the Luminex™ 200 system, and humoral immune responses against tachyzoite and MNC antigens were compared by ELISA, Western blotting and immunoproteomics. Our results showed that BKI-1294 treatment of live-vaccinated mice reduced the cerebral parasite load in the dams, but resulted in higher neonatal pup mortality and vertical transmission. In live-vaccinated mice, cytokine levels, most notably IFN-y, IL-10, and IL-12, were consistently lower in BKI-1294 treated animals compared to non-treated mice. In addition, comparative Western blotting identified two protein bands in MNC extracts that were only recognized by sera of live-vaccinated mice treated with BKI-1294, and were not found in tachyzoite extracts. We conclude that treatment of live-vaccinated mice with BKI-1294 influenced the cellular and humoral immune responses against infection, affected the safety of the live-vaccine, and decreased protection against re-infection and vertical transmission during pregnancy.

## Introduction

The apicomplexan parasite *Neospora caninum* is an important causative agent of abortion or birth of weak offspring in cattle, and to a lesser extent in sheep and other ruminants (1). The sexual cycle of *N. caninum* takes place in canine intestinal tissue, and dogs do not only shed oocysts which become infective after sporulation, but can also act as intermediate hosts and become affected by neurological symptoms. Rapidly proliferating tachyzoites represent the disease-causing stage that can cross the placenta and infect the fetus. Upon infection of an immunocompetent host, differentiation into tissue-cyst-forming bradyzoites takes place, which remain viable, proliferate slowly, and do not cause inflammation. Infection of fetuses during pregnancy occurs via exogenous transplacental transmission in cases where primary maternal infection takes place during the course of the pregnancy, or endogenous transplacental transmission in cases where persistently infected animals undergo pregnancy-driven immunomodulation that leads to recrudescence and bradyzoite-tachyzoite reconversion (2, 3). Currently there is no vaccine on the market for the prevention of bovine or canine neosporosis and so far, no immuno- or chemo-therapeutic treatments have found their way to the market. However, live-vaccines comprised of low-virulence strains of *N. caninum* strains have shown promising efficacy in both murine and bovine models (4–8).

Protein kinases are involved in functional activities that control essential aspects of apicomplexan biology, including host cell invasion, intracellular proliferation, and egress (9–11). Calcium-dependent protein kinases (CDPKs) are especially interesting drug targets in that homologs of CDPKs are not found in mammals. One of these kinases is CDPK1, homologs of which are intensively studied in target-based drug development for a wide range of apicomplexans including *Plasmodium, Cryptosporidium, Eimeria, Babesia, Theileria, Toxoplasma*, and *Neospora* among others [reviewed by (12, 13)]. In *N. caninum* and *T. gondii*, CDPK1 is essential for motility, host cell invasion, and egress of tachyzoites from the host cell, through its role in the signaling events that control microneme secretion (14, 15).

Bumped kinase inhibitors (BKIs) are ATP-competitive CDPK1 inhibitors. They exhibit a high degree of efficacy and specificity for apicomplexan CDPK1, as they are optimized to fit their C-3 bulky aromatic substituents into a hydrophobic pocket behind the atypically small gatekeeper residue. This large hydrophobic pocket is absent in most mammalian kinases ATP binding sites (16). The specificity of many BKIs is further mediated by binding to the hinge region of the active site of CDPK1, similar to the binding motif of ATP while maintaining additional hydrogen bond interaction with residues in the ribose pocket (17, 18). Several BKIs have been studied so far with respect to efficacy against *N. caninum* infection, including the pyrazolopyrimidine BKI-1294. BKI-1294 interferes with host cell invasion and egress, but does not act parasiticidal *in vitro* (19). BKI-1294 also affects the intracellular tachyzoites by inducing the formation of multinucleated complexes (MNCs) (20), which are characterized by continued nuclear division, and the formation of intracellular zoites separated by the inner membrane complex, but lacking the major surface antigen 1 (SAG1) (21). However, these zoites failed to undergo disjunction and tachyzoite formation, remained trapped within the host cell cytoplasm, and exhibited altered mRNA and protein expression (19, 22). MNCs remained viable for up to 20 days under constant BKI-1294 drug pressure *in vitro*, and upon drug removal the block in zoite disjunction was alleviated and new infective tachyzoites were formed, which resumed proliferation (21).

Nevertheless, BKI-1294 is very effective *in vivo*, and in pregnant mice treatment strongly increased pup survival and inhibited exogenous transplacental transmission of *N. caninum* (19) in an efficient manner. BKI-1294 was also efficacious in pregnant mice and in pregnant sheep infected with the closely related *Toxoplasma gondii* (23, 24). Other MNC-inducing BKIs such as BKI-1517 and BKI-1553 were also effective against neosporosis, as shown in pregnant mouse and sheep models (25, 26). All these earlier studies focused on treatments to prevent exogenous transplacental transmission of *N. caninum*. We here show that BKI-1294 therapy in live-vaccinated, thus already infected, mice has a negative impact on the safety and the protective activity against challenge infection during pregnancy, and this is accompanied by alterations in cellular and humoral immune responses in live-vaccinated mice. Thus, the use of BKI-1294 in *N. caninum* infected animals could have detrimental effects upon re-infection during pregnancy.

## Materials and Methods

### Tissue Culture and Media, Biochemicals, and Drugs

If not stated otherwise all tissue culture devices were purchased from Sarstedt (Sevelen, Switzerland), culture media were from Gibco-BRL (Zürich, Switzerland) and biochemicals were from Sigma (St. Louis, MO). Molecular biology kits were obtained from Qiagen (Hilden, Germany). BKI-1294 was synthesized to >99% purity by NMR and HPLC by Wuxi AppTec (Shanghai, China), in a method that was previously described (27). For *in vitro* treatments, BKI-1294 was stored as a 20 mM stock solution in dimethyl sulfoxide (DMSO) at −20°C; for application in mice, BKI-1294 powder was suspended in corn oil, and the suspension was applied by gavage.

### Host Cell and Parasite *in vitro* Culture

Human foreskin fibroblasts (HFF; ATCC® SCRC-1041™) and BALB/c dermal fibroblasts (ATCC® CCL-163) were maintained in Dulbecco's modified Eagle medium (DMEM, high glucose, GlutaMAX™ Supplement, HEPES), and the monkey kidney cell line Marc-145 (ATCC® CRL-12231) was cultured in DMEM without sodium pyruvate and HEPES. Media were supplemented with phenol red, 10% heat-inactivated and sterile filtered fetal calf serum (FCS), 50 U of penicillin/ml, and 50 μg streptomycin / ml. The *N. caninum* isolates Nc-Liverpool (NC-Liv) and Nc-Spain7 (Nc-Sp7) were maintained by infecting semi-confluent HFF or Marc-145 monolayers and culture at 37°C/5% CO_2_, with passages once or twice per week. To obtain tachyzoites for infection of BALB/c mice, infected monolayers were washed in medium without serum, and scraped from tissue culture flasks when tachyzoites were still largely intracellular (>90% of undisrupted parasitophorous vacuoles). They were repeatedly passed through a 25-gauge needle, liberated parasites were suspended in 0.2% trypan blue in PBS and counted in a hemocytometer. They were used for infection experiments when at least >95% viable.

### Ethical Statement

Animal studies were approved by the Animal Welfare Committee of the Canton of Bern under the license BE96/117. BALB/c mice were purchased from Charles River (Sulzberg, Germany), and were housed in a common room, under controlled temperature and a 14 h dark/10 h light cycle, with food and water ad libitum, according to guidelines of animal welfare legislation of the Swiss Veterinary Office. The animals were handled in strict accordance with practices to minimize suffering.

### Live-Vaccination and BKI-1294 Treatments, and Re-infection of BALB/c Mice During Pregnancy

Eighty-eight (88) female and 44 male BALB/c mice, 6–8 weeks of age, were used in the experiment. Females were randomly assigned to 6 experimental groups (treatment groups A-E: 16 females/group). The experimental treatments for each group are summarized in Table 1, and a timeline for all treatments is shown in Supplementary Figure 1. On day 0, 4 groups (A–D) were live-vaccinated by subcutaneous (s.c.) injection of 10^5^ Nc-Liv tachyzoites, while groups E and F remained uninfected and mock-vaccinated with PBS. Groups B and C received BKI-1294 treatments (50 mg/kg in corn oil by gavage) on a daily basis for 5 days, starting on day 2 post-live-vaccination, the other groups received corn oil only. All animals were mated during days 21–23, with one male assigned to two females. Groups C, D, and E were subsequently re-infected on day 28, by s.c. inoculation of 2 × 10^5^ Nc-Sp7 tachyzoites (28, 29). After separation of pregnant from non-pregnant mice on day 36 post-infection (p.i.), dams were put into individual cages and gave birth on days 40–42 post-infection (p.i.). They were allowed to rear their pups and were closely monitored for 30 days. On day 72, all surviving animals were euthanized in a chamber floated with the anesthetic isoflurane, followed by CO_2_. The brain was dissected and frozen at −80°C for subsequent determination of the cerebral parasite burden. Blood was collected at 3 timepoints during this experiment: from the tail vein 14 days post live-vaccination and on day 16 of pregnancy, and by heart puncture on day 30 post-partum.

**Table 1:** Experimental groups for the assessment of BKI-1294 treatment effects in *N. caninum* infected mice, mated and re-challenged or not during pregnancy.

**Groups^**a**^**	**Live vaccine**	**BKI-1294**	**Mating/pregnancy**	**Re-challenge**
	**day 0^**b**^**	**d2-7^**c**^**	**d21-23**	**day 28^**d**^**
**A** (v, t, i)	Yes	yes	yes	Yes
**B** (v, t, –)	Yes	yes	yes	No
**C** (v, –, i)	Yes	no	yes	Yes
**D** (v, –,–)	Yes	no	yes	No
**E** (–, –, i)	No	no	yes	Yes
**F** (–, –, –)	No	no	yes	No

a *v, vaccinated; t, BKI-1294 treated; i, infected during pregnancy; n = 16 for all groups*.

b *s.c. infection with 1 × 10^5^ Nc-Liv tachyzoites*.

c *Daily application by gavage of BKI-1294, 50 mg/kg/day during 5 days*.

d *s.c. infection with 2 × 10^5^ Nc-Sp7 tachyzoites during pregnancy*.

### Determination of the Cerebral Parasite Burden by Real Time PCR

The cerebral parasite burden of dams and surviving pups was analyzed by *N. caninum*-specific real-time PCR of brain tissue DNA as previously described (29, 30). DNA extraction was performed using the Nucleospin Kit (Macherey-Nagel, Oensingen, Switzerland). The DNA concentration was determined using the QuantiFluor dsDNA System (Promega, Madison, Wi., USA) and was adjusted to 5 ng/ml with sterile DNase free water. The sequence of the forward primer P-NEO-PF1 was 5′-CCCAGTGCGTCCAATCCTGTAAC-3′, the sequence of the reverse primer P-NEO-PR1 was 5′-CTCGCCAGTCAACCTACGTCTTCT-3′. Fluorescent probes were P-NEO-SO3FL (5′-CACGTATCCCACCTCTCACCGCTACCA-3′) and P-NEO-SO5LC (5′-TCCCTCGGTTCACCCGTTCACACAC-3′). Quantitative real-time PCR was performed using the Rotor-Gene 6000 real-time PCR machine. The parasite load was calculated by interpolation from a standard curve with DNA equivalents from 1,000, 100, and 10 *N. caninum* tachyzoites included in each run.

### Preparation of *N. caninum* Tachyzoite- and MNC-Extracts

Tachyzoite and MNC preparations were done essentially as described (22). In short, semi-confluent BALB/c dermal fibroblast monolayers were maintained in T175 cell culture flasks infected with 10^6^ Nc-Liv tachyzoites and passaged at least twice prior to the experiment. At 4 h post infection, treatment with 5 μM BKI-1294, or the corresponding amount of DMSO in controls, was initiated. Cultures were maintained at 37°C/5% CO_2_ during 3 days for generating tachyzoites as DMSO-treated controls, or during 6 days in the presence of BKI-1294 for generating MNCs. Subsequently, the infected monolayers were washed twice with PBS, followed by removal of infected cells with a rubber cell scraper and resuspension in PBS. After passaging the suspensions twice through a 25-gauge needle to break the host cells. MNCs and tachyzoites were separated from host cell debris by Sephadex G-25 chromatography as described (31). The parasite fractions in the flow through were collected by centrifugation (15 min, 1,000 × g, 4°C) and washed twice with PBS. The protein content was measured with a Pierce™ BCA Assay Kit (ThermoFisher Scientific, Reinach, Switzerland) and samples were stored at −80°C.

### Analysis of IgG1 and IgG2 Responses by Enzyme Linked Immunosorbent Assay (ELISA)

The levels of IgG1 and IgG2a were measured by ELISA as previously described (32). Briefly, 96-well-plates were coated overnight at 4°C with 200 ng of *N. caninum* tachyzoite extract in 100 μl coating buffer per well. The extract was obtained by subjecting tachyzoites to three freeze–thaw cycles and sonication for 4 x 30 s at 57 W before being filtered through a 20 μm sterile filter. After three washes, plates were blocked with 1% bovine serum albumin in wash buffer [PBS with 0.05% Tween-20 (v/v)] and serum samples (4 replicates per sample) were applied at room temperature (RT). After three washes, plates were incubated with either goat anti-mouse IgG1 or IgG2a conjugated to alkaline phosphatase (AP) (SouthernBiotech, Birmingham, USA). The reaction was developed with AP substrate and absorbance values as optical density (OD) at 405 nm read in a tunable microplate reader (EnSpire™ 2300 Multilabel Reader, Switzerland). In order to compare OD values between samples analyzed in different plates, the same positive and negative serum controls were added in each plate and OD values for each sample were converted into a relative index per cent (RIPC) using the following formula RIPC = (OD405 sample-OD405 negative control)/(OD405 positive control-OD405 negative control) (33).

### Measurements of the Direct Impact of BKI-1294 on Splenocyte Proliferation

Splenocyte proliferation assays were carried-out as previously described (34). Briefly, splenocytes were isolated from female BALB/c mice and were distributed in polystyrene 96 well-flat bottom plates at 2 × 10^5^ cells/100 μL/well. Splenocytes were either left unstimulated or were stimulated with Concanavalin A (ConA, 5 μg/mL), lipopolysaccharide (LPS, 10 μg/mL), ConA plus 0.4 μM BKI-1294 or 0.3 μM pyrimethamine (Pyr), or LPS plus 0.4 μM BKI-1294 or 0.3 μM Pyr. Each assay was done in quadruplicates. Cultures were maintained in a 37°C humidified chamber containing 5% CO_2_ for a total incubation period of 72 h. Proliferation of splenocytes was measured using a 5-bromo-20-deoxy-uridine (BrdU) cell proliferation kit (QIA58, Merck Millipore). Data are presented as mean ± standard deviation (SD) for the indicated numbers.

### Measurements of Cytokine Levels in Sera and in Splenocyte Culture Supernatants by Multiplex Immunoassay

BALB/c mice (6 animals/group) were live-vaccinated by s.c. inoculation of 10^5^ Nc-Liv tachyzoites as above, and treated with BKI-1294 or not, as described in above. Blood samples (max. 100 μl) were collected from the tail vein 4 days p.i (after 2 days of treatment), 11 days p.i. (5 days post-treatment stop), and by cardiac puncture at 26 days p.i. (20 days post treatment-stop) following euthanasia. Splenocytes obtained after euthanasia were cultured *in vitro* and were stimulated with *N. caninum* tachyzoite extract or remained unstimulated, and medium supernatants were collected after 72 h and stored at −80 °C. Luminex xMAP technology (35) was used for determination of cytokines, chemokines, and growth factors from pooled medium and serum samples from the 6 animals. The multiplexing analysis was performed using the Luminex™ 200 system (Luminex, Austin, TX, USA) by Eve Technologies Corp. (Calgary, Alberta). Measurements were done using Eve Technologies' Mouse Focused 10-Plex Discovery Assay® (MilliporeSigma, Burlington, Massachusetts, USA) according to the manufacturer's protocol. The 10-plex consisted of GM-CSF, IFNγ, IL-1β, IL-2, IL-4, IL-6, IL-10, IL-12 (p70), MCP-1, and TNFα. Assay sensitivities of these markers range from 0.4 to 10.9 pg/ml. Individual analyte sensitivity values are available in the MilliporeSigma MILLIPLEX® MAP protocol.

### SDS-PAGE and Western Blotting

BALB/c mice, 4 animals per group, were live-vaccinated as above, and treated with BKI-1294 (50 mg/kg/day for 5 days) or with corn oil alone as described above. Mice were euthanized on day 26 p.i., and blood was collected by heart puncture. The sera of each group were pooled and stored at −20°C prior to use. Tachyzoite and MNC fractions (see above) were taken up in SDS-sample buffer to reach a protein concentration of 1 μg/ml, and 20 μl of each extract was separated by preparative 8% SDS-PAGE, using a Hoefer Minigel 250 Apparatus (GE Healthcare, Little Chalfont, UK). Gels were either silver stained (36), or Western blotting onto polyvinylidene difluoride membrane was carried out using a Hoefer TE22 Mighty Small Transphor, for 1 h at 100 V (21). The membrane was cut vertically into small strips, and selected strips were blocked for 1 h at RT in PBS/0.1% Tween-20/2% milk powder, while the remaining strips were air-dried and stored at RT in the dark. The blocked strips of each fraction were then incubated with serum from live-vaccinated + BKI-1294 treated mice or non-treated mice (both diluted 1:100 in blocking buffer, 15 h, 4°C). Following washes in PBS-Tween, the strips were incubated with anti-mouse IgG (H&L) Alkaline Phosphatase (AP) conjugate (Promega, Madison, USA) at a dilution of 1:2,000 in blocking solution for 1 h at RT. Bound antibodies were visualized by a color reaction using 0.3% BCIP/NBT stock solution (Roche, Germany) in AP development buffer (0.1 M NaCl, 5 mM MgCl_2_, and 0.1 M Tris pH 9.5). Prior to drying at RT and alignment of the strips, they were washed extensively with H_2_O.

### Immunoproteomics

In order to identify proteins that are present in bands specifically recognized in MNC extract, strips were carefully aligned and the specifically labeled bands in MNC extracts, as well as the non-labeled areas of identical molecular mass in tachyzoite extracts, were cut out and their protein composition was analyzed at the Mass Spectrometry and Proteomics Facility, Department of Clinical Research, University of Bern. For this, each band was further cut into fragments of 1 mm^2^, blocked in 0.5% PVP-40, reduced, alkylated and digested with trypsin. The digests were analyzed by liquid chromatography (LC)-MS/MS (PROXEON coupled to a QExactive HF mass spectrometer, ThermoFisher Scientific) with one injection of 5 μl digests. Peptides were trapped on a μPrecolumn C18 PepMap100 (5 μm, 100 Å, 300 μm × 5 mm, ThermoFisher Scientific) and separated by backflush on a C18 column (5 μm, 100 Å, 75 μm × 15 cm, C18) by applying a 20-min gradient of 5% acetonitrile to 40% in water, 0.1% formic acid, at a flow rate of 300 nl/min. The Full Scan method was set with resolution at 60,000 with an automatic gain control (AGC) target of 1E06 and maximum ion injection time of 50 ms. The data-dependent method for precursor ion fragmentation was applied with the following settings: resolution 15,000, AGC of 1E05, maximum ion time of 110 milliseconds, mass window 1.6 m/z, collision energy 27, under fill ratio 1%, charge exclusion of unassigned and 1+ ions, and peptide match preferred, respectively. Spectra interpretation was performed with Easyprot on a local server run under Ubuntu against a forward + reverse *Neospora caninum* Liverpool database, using fixed modifications of carboamidomethylated on Cys, and variable modification of oxidation on Met, and acetylation on protein N-Term. Parent and fragment mass tolerances were set to 10 ppm and 0.4 Da, respectively. Matches on the reversed sequence database were used to set a Z-score threshold, where 1% false discoveries on the peptide spectrum match level had to be expected. Protein identifications were only accepted, when two unique peptides fulfilling the 1% FDR criterium were identified.

### Statistics

The percentages of survivors and vertical transmission at the end of the experiment or percentages of *N. caninum* PCR positive samples were analyzed by Chi-square test with Yates' continuity correction in a contingency table. Statistical analysis of the parasite burdens in brains was done using the Kruskal–Wallis test followed by the Wilcoxon rank-sum test with Holm adjustment. Differences in humoral immune responses were assessed by Kruskal-Wallis or U Mann-Whitney test.

## Results

### Effects of BKI-1294 Treatment in Live-Vaccinated Mice Re-infected With *N. caninum* Tachyzoites During Pregnancy

The structure to study the impact of BKI-1294 treatment in live-vaccinated mice consisted of 6 groups (A–F) and is shown in Table 1. The outcome of this study is summarized in Table 2. Groups A–D were live vaccinated with an infection dose of 10^5^ Nc-Liv tachyzoites, and none of the mice exhibited clinical signs or succumbed to disease during the entire experiment. The live-vaccinated groups (A–D) exhibited similar fertility rates (50–80%) as the non-infected mice (groups E and F). Litter sizes ranged between 4.9 and 5.8 pups/dam. Live vaccination without further treatments as shown in group D led to survival of all pups, and no vertical transmission occurred, demonstrating the safety of this live vaccination approach. However, treatment with BKI-1294 following live vaccination (group B) resulted in 12.5% neonatal mortality, but postnatal mortality was restricted to only 1 pup out of 56, and vertical transmission occurred in 6 out of 56 pups. As can be seen in group C, live vaccination was also very efficacious in terms of protection against re-infection during pregnancy, with neonatal mortality detected in only 2 out 57 pups, but no cases of postnatal mortality, and also no vertical transmission. In contrast, BKI-1294 treatment (group A) decreased protection against re-infection during pregnancy, especially with regard to vertical transmission, as it was detected in over 21% (8 out of 37) of the pups, which was significantly higher than in group C (*P* < 0.05). The highest rates of neonatal and postnatal mortality were seen in group E, which was only challenge-infected during pregnancy. Overall, applying BKI-1294 treatment following live-vaccination lead to a partial deterioration of the safety as well as the efficacy of the live vaccine.

**Table 2:** Outcome of *N. caninum* infection in live-vaccinated and/or non-vaccinated pregnant mice, treated or not with BKI-1294 prior to challenge infection during pregnancy.

**Group^**a**^**	**Fertility (%)^**b**^**	**Litter size (pups/dam)^**c**^**	**Neonatal mortality (%)^**d**^**	**Postnatal mortality (%)^**e**^**	**Postnatal survival (%)^**f**^**	**Vertical transmission (%)^**g**^**
**A** (v; t, i)	8/16 (50)	39 (4.9)	2/39 (5.1)	3/37 (8.1)	34/37 (91.9)	8/37 (21.6)
**B** (v, t, –)	13/16 (80)	64 (4.9)	8/64 (12.5)	1/56 (1.8)	55/56 (98.2)	6/56 (10.7)
**C** (v, –, i)	11/16 (70)	57 (5.1)	2/57 (3.5)	0/55 (0)	55/55 (100)	0/55 (0)
**D** (v, –, –)	12/16 (75)	69 (5.8)	0/69 (0)	0/69 (0)	69/69 (100)	0/69 (0)
**E** (–, –, i)	12/16 (75)	58 (4.8)	8/58 (13.8)	46/50 (92)	4/50 (8)	48/50 (96)
**F** (–, –, –)	8/16 (50)	40 (5)	2/40 (5)	0/38 (0)	38/38 (100)	0/38 (0)

a *v, vaccinated; t, BKI-1294 treated; i, infected during pregnancy*.

b *Proportion of pregnant mice per group (%)*.

c *Number of delivered pups per dam*.

d *Proportion of pups born dead or that died within the first 2 days post-partum (%)*.

e *Proportion of pups died from day 3 to 30 post-partum (%)*.

f *Proportion of survival pups at day 30 post-partum (%)*.

g *Proportion of Neospora caninum-PCR positive surviving pups plus those which died from day 3 post-partum [dead pups from day 3 post-partum are considered N. caninum-PCR positive as previously shown (37)]*.

**Figure 1:**
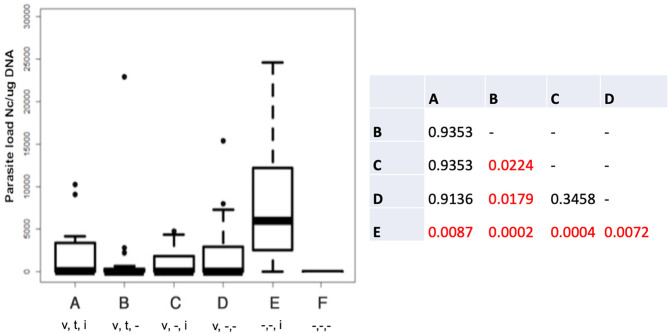
Cerebral parasite loads in the dams of the different treatment groups measured by quantitative real time PCR and presented as box plots. The table shows the results of the statistical evaluation, *p*-values in red indicate significant differences; *v* = vaccinated; *t* = BKI-1294 treated; *i* = infected during pregnancy; - indicates no treatment; *n* = 16 for all groups.

The cerebral parasite load in the dams at 30 days post-partum was determined by quantitative real time PCR (Figure 1), and was significantly elevated in group E compared to all other groups. In group B (live vaccinated and BKI-1294 treated without re-infection), cerebral parasite loads in the dams were significantly lower compared to those groups that were not BKI-1294 treated, namely group C (live-vaccinated and then re-infected), and group D (only live-vaccinated).

**Figure 2:**
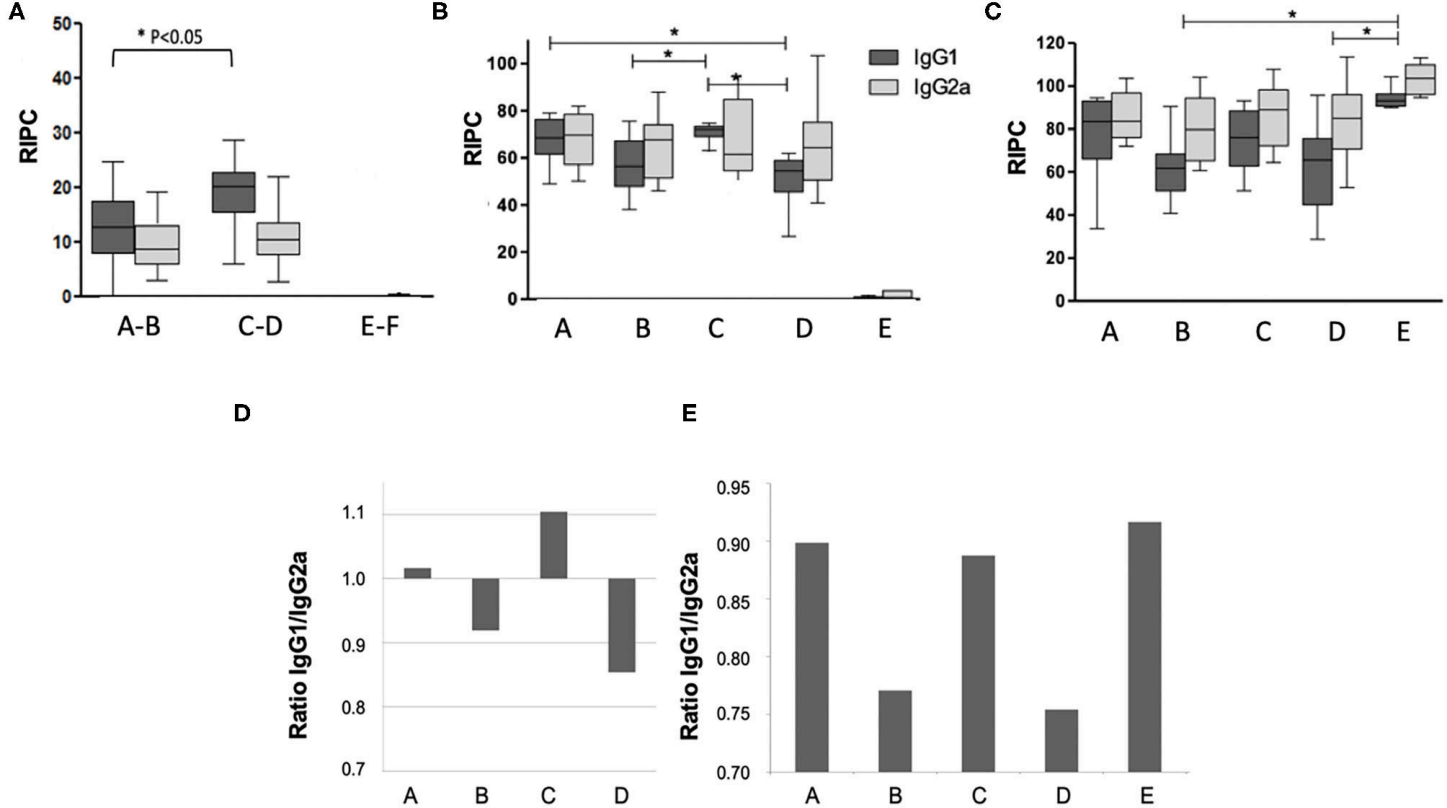
Humoral immune responses in the pregnant mice of the different treatment groups A–E. Group F is omitted, since these animals were not infected. Blood was collected at 14 days post-live vaccination **(A)**, on day 15 of pregnancy = day 8 post-re-challenge infection **(B)**, and on day 30 post-partum = 40 days after re-challenge **(C)**. In panel A, groups A and B (both live-vaccinated and treated), C and D (both live-vaccinated but not treated) and E and F (both non-vaccinated and not treated) were grouped together, as at this time point animals from these groups had undergone the same treatments. Antibody levels on the y-axis are indicated as relative index per cent (RIPC) values) * indicates *P* < 0.05. **(D,E)** Show IgG1/IgG2a ratios in the different treatment groups at day 15 of pregnancy **(E)** and day 30 post-partum (F).

Antibody responses against *N. caninum* antigens were assessed by ELISA at three timepoints (Figure 2): (i) 14 days post live-vaccination and 12 days post-BKI-1294 treatment); (ii) on day 16 of pregnancy; and (iii) on day 30 post-partum. Antibody levels measured prior to pregnancy (Figure 2A) were below those at the later timepoints. However, IgG1 levels in groups A-B (treated with BKI-1294) were significantly lower compared to groups C-D (non-treated), while IgG2a levels were similar. At 16 days of pregnancy, 8 days after the mice had been re-challenged (Figure 2B), groups A and C, both live vaccinated and re-challenged, exhibited higher IgG1 levels compared to groups B and D, which had been only live-vaccinated but were not re-infected during pregnancy, indicating that re-infection induced an antibody response. No significant difference in IgG1 levels could be seen in the corresponding BKI-1294 treated groups A and B, and no antibody response could be measured in group E at this timepoint. However, at 30 days post-partum (Figure 2C), IgG1 and IgG2 levels in group E (challenged only once during pregnancy) were significantly higher compared to groups B and D, which both were only live vaccinated, either with (group B) or without (group D) subsequent BKI-1294 treatment. A comparison of the IgG1/IgG2a ratios at day 16 of pregnancy (Figure 2D) revealed that those groups that were live-vaccinated and re-infected (groups A and C) exhibited a predominantly IgG1-biased response, which was much less pronounced in group A (treated with BKI-1294 following live vaccination). In contrast, those groups that were only live-vaccinated without re-infection (B and D), exhibited IgG2a-biased responses, independent of treatment. On day 30 post-partum (Figure 2E), the IgG1/IgG2a ratios in groups A and C were more balanced, similar to group E, and in group B and D (only live-vaccinated) the IgG2a-bias was maintained (Figure 2E).

### Effects of BKI-1294 Treatment on the Cellular Immune Response in Live-Vaccinated Mice

**Figure 3:**
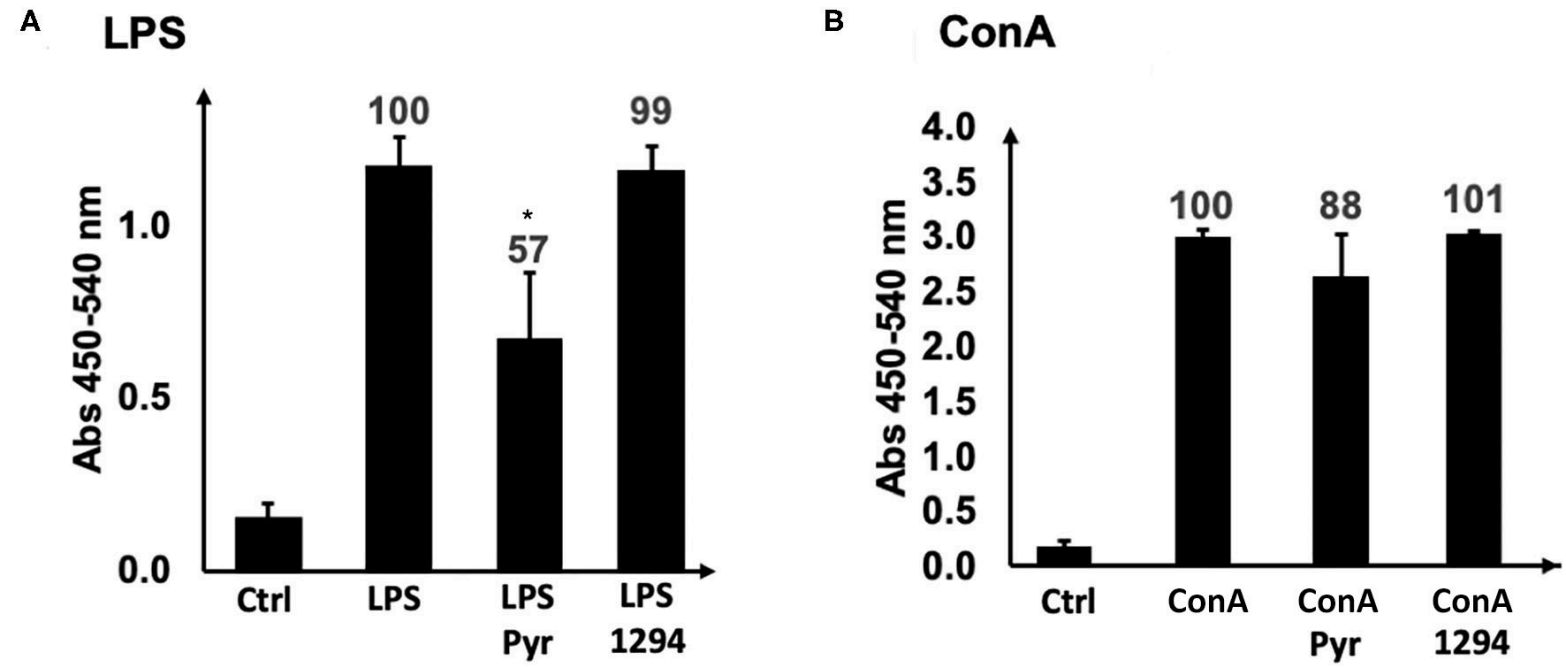
Direct effects of BKI-1294 and pyrimethamine on proliferation of LPS- and ConA treated murine splenocytes. Bars represent standard deviation from the mean of four replicates. A 100% of proliferation is attributed to controls (ConA and LPS); values indicate percent of proliferation compared to control (*) *p* < 0.01 in relation to controls (ConA or LPS). Proliferation **(A,B)** was assessed by BrdU incorporation.

We first investigated whether BKI-1294 itself could have a direct impact on the proliferative capacities of B- or T- cells. For this, naïve BALB/c mouse splenocytes were isolated and cultured *in vitro* and were treated with ConA (for T cell stimulation) or LPS (for B cell stimulation), and 0.4 μM BKI-1294 or 0.3 μM pyrimethamine were added concomitantly to the mitogens. After an additional 72 h in culture, no inhibitory effect on ConA or LPS-induced proliferation of splenocytes was visible in BKI-1294 treated cultures, while pyrimethamine inhibited the proliferation of B cells by almost 50% (Figure 3).

Subsequently, the potential impact of BKI-1294 treatment on the cellular immune response in mice live-vaccinated with *N. caninum* was studied by assessing cytokine levels in medium supernatants of splenocytes cultured with or without stimulation with crude *N. caninum* antigen extract (Nc-antigen). For this, live-vaccinated BALB/c mice were treated either with BKI-1294 emulsified in corn oil, or corn oil alone, during 5 days, starting at 2 days p.i. None of the animals showed any clinical signs throughout the observation period. Following euthanasia splenocytes from each treatment group were cultured *in vitro* and were stimulated with Nc-antigen or remained unstimulated. Medium supernatants were collected after 3 days, and subjected to cytokine analysis. The results are summarized in Table 3. Medium supernatants obtained from non-stimulated control splenocyte cultures showed consistently lower cytokine levels compared to Nc-antigen stimulated cultures. The highest concentrations by far were measured for IFN-γ, which were higher in Nc-antigen stimulated splenocytes of non-treated mice compared to BKI-1294 treated mice. For splenocyte cultures of live-vaccinated mice that were not BKI-1294 treated, cytokine concentrations were higher, with the exception of GM CSF, IL-2, IL-6, and TNF-alpha.

**Table 3:** Cytokine levels (pg/ml) measured in medium supernatants of splenocytes obtained from live-vaccinated mice on day 26 p.i., as determined by multiplex immunoassay.

		**IFNy**	**IL-1b**	**GM CSF**	**IL-2**	**IL-4**	**IL-6**	**IL-10**	**IL-12**	**MCP-1**	**TNFa**
Nc-antigen	+BKI-1294	50000^*^	24.4	1089.6	272.8	455.3	1231.2	3521.2	22.8	557.8	169.7
Nc-antigen	–BKI-1294	70000^*^	25.6	866.6	216.6	818.3	979.8	4373.7	31.4	730.2	139.9
non-stim	+BKI-1294	1354.8	9.1	686.5	1132.9	87.4	373.0	155.8	4.4	540.5	29.0
non-stim	–BKI-1294	1077.9	9.1	709.0	1217.5	79.8	286.9	118.3	21.5	602.2	20.7

*Live-vaccinated mice were either treated by oral application of BKI-1294 (50 mg/kg/day for 5 days), starting on day 2 p.i. Individual values are from 6 pooled medium supernatants of splenocyte cultures that were either stimulated with N. caninum tachyzoite extract (Nc-antigen, 10 μg/ml) or remained non-stimulated (non-stim)*.

* *The concentrations of IFN-y are exceedingly high and estimated values are shown. Measurements were done on pooled samples from 6 mice, using Eve Technologies' Mouse Focused 10-Plex Discovery Assay® (MilliporeSigma, Burlington, Massachusetts, USA)*.

**Figure 4:**
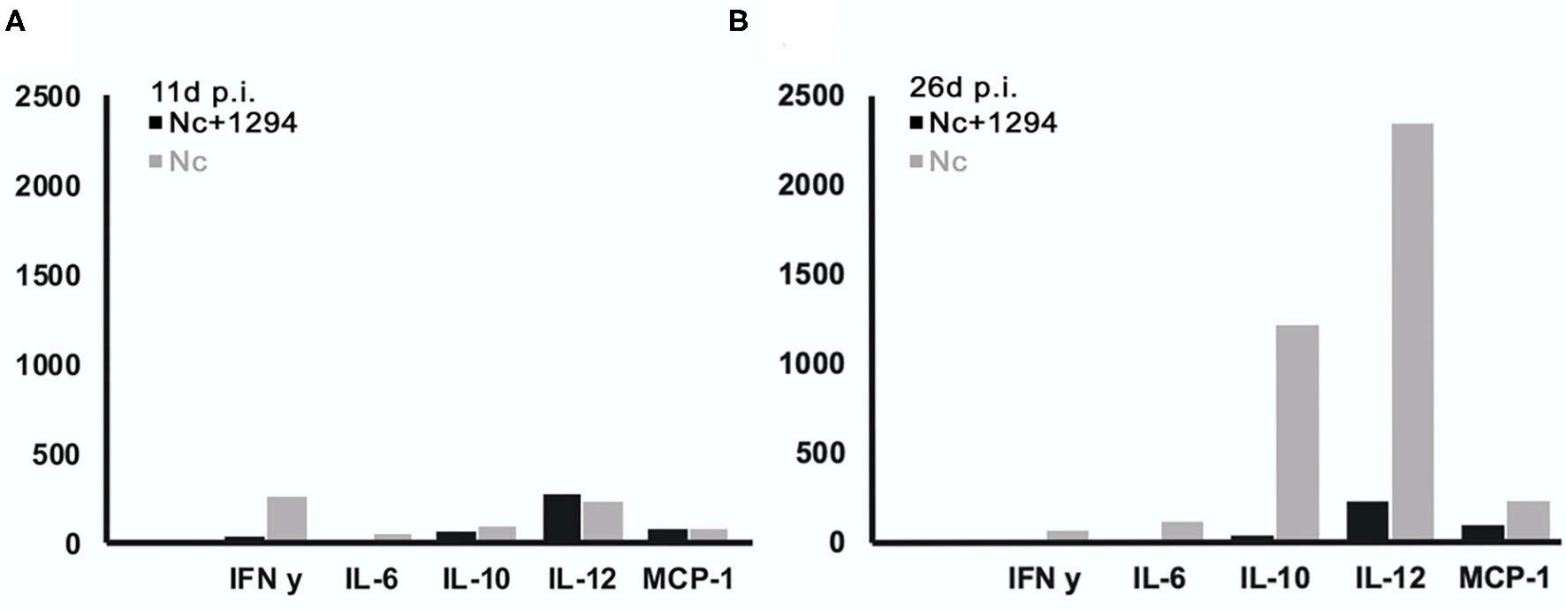
Cytokine levels (pg/ml) measured in sera of *N. caninum* infected mice and control mice, treated or not with BKI-1294. Blood samples were collected from the tail vein at day 11p.i. **(A)** and day 26 p.i. **(B)**.

Cytokine levels were also determined in serum samples collected from *N. caninum* infected untreated and BKI-1294 treated BALB/c mice at 4, 11, and 26 days p.i. (corresponding to day 2 of treatment and days 4 and 21 post-treatment, respectively). The results for selected cytokines on days 11 and 26 are shown in Figure 4, and values for all cytokines at all timepoints are shown in Table 4. At 4 days p.i., cytokine levels were still low in both groups (max 34 pg/ml), thus no differences were noted between live-vaccinated and BKI-1294-treated and non-treated mice. More pronounced changes were seen on day 11 p.i., (Figure 4A), with 5–6 times higher IFN-γ levels in the placebo treated / live-vaccinated group (253 pg/ml) compared to the BKI-1294-treated / infected group (42 pg/ml). However, elevated IL-10 (67–94 pg/ml), IL-12 (235–278 pg/ml), and MCP-1 levels (83–86 pg/ml) were also noted in all 4 groups at that intermediate timepoint. On day 26 p.i. (Figure 4B), IFN-γ was diminished, while IL-2 and IL-6 were more clearly discernible, especially in sera of non-treated mice. The predominant cytokines were IL-10 and IL12, both at much higher levels (1,215 and 2,341 pg/ml, respectively) in the placebo-treated live vaccinated mice, compared to the live-vaccinated and BKI-1294 treated mice (38 and 230 pg/ml, respectively). In most instances the sera of live vaccinated and BKI-1294 treated mice exhibited lower cytokine levels compared to non-treated mice (see Table 4).

**Table 4:** Serum cytokine levels (pg/ml) of live-vaccinated mice (pooled samples of 6 mice) taken on day 4 (d4.p.i.), day 11 (d11.p.i.), and day 26 (d26p.i. 26 p.i.), as determined by multiplex immunoassay.

		**IFN y**	**IL-1B**	**GM CSF**	**IL-2**	**IL-4**	**IL-6**	**IL-10**	**IL-12**	**MCP-1**	**TNFa**
4d.p.i	+BKI-1294	0	0	0	0	0	13.1	1.5	0	52.2	5.6
4d.p.i.	–BKI-1294	0	0	0	0	0.17	9.3	3.6	0	34.9	5.6
11d.p.i.	+BKI-1294	42.2	0.0	6.3	5.5	4.1	14.3	67.7	277.9	85.9	11.9
11d.p.i.	–BKI-1294	253.7	1.4	6.3	10.9	12.3	46.7	94.3	234.8	83.2	16.4
26d.p.i.	+BKI-1294	15.2	15.0	6.3	71.4	1.2	7.5	38.0	229.8	99.3	10.3
26d.p.i.	–BKI-1294	70.4	97.8	22.7	200.8	38.4	116.7	1215.1	2340.9	231.5	17.6

*Live-vaccinated mice were either treated by oral application of BKI-1294 (50 mg/kg/day for 5 days), starting on day 2 p.i. Measurements were done using Eve Technologies' Mouse Focused 10-Plex Discovery Assay® (MilliporeSigma, Burlington, Massachusetts, USA)*.

### Effects of BKI-1294 on the Antibody Recognition Pattern in Live-Vaccinated Mice

**Figure 5:**
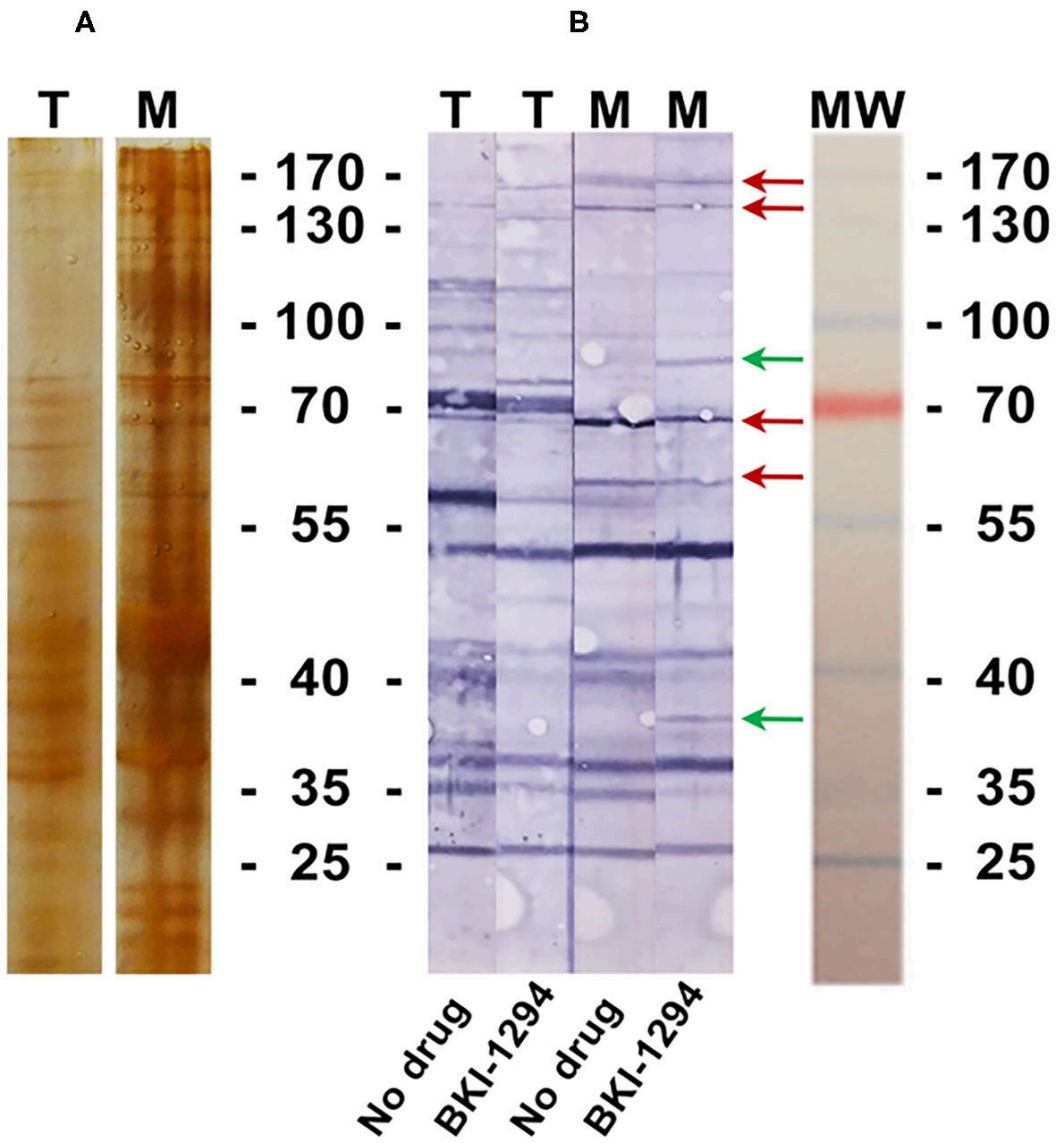
Silver stained SDS-PAGE **(A)** and Western blot **(B)** of tachyzoite (T) and MNC (M) extracts, labeled with sera from live-vaccinated mice that were either not treated (No drug) or with sera from live-vaccinated mice treated with BKI-1294 (BKI-1294). Red arrows indicate bands that are stained in MNC extract by both antisera, green arrows are those bands labeled with serum from BKI-1294 treated mice only.

BALB/c mice were live-vaccinated with *N. caninum* tachyzoites and were treated either with BKI-1294 emulsified in corn oil or with corn oil alone for 5 days, starting at 2 days p.i. At 26 days p.i., mice were euthanized and serum samples were obtained and pooled for each group. None of the animals showed any clinical signs during this period. For Western blot analysis, MNCs and tachyzoite extracts were separated by SDS-PAGE and visualized by Silver staining (Figure 5A). The reactivities of the antisera obtained from placebo-treated and BKI-1294 treated mice were assessed by Western blotting (Figure 5B). Alignment of the strips showed that the overall banding patterns in all blots were similar. However, upon closer inspection we identified 6 bands that were stained only in blots of MNC extracts, and were absent or largely diminished in tachyzoite blots. This led to the identification of 4 bands in MNC extracts that were recognized by both sera (red arrows, Figure 5B), and two bands of 90 and 38 kDa (green arrows, Figure 5B) that were labeled only by sera from BKI-1294 treated mice. These MNC-associated bands could not be detected in extracts of non-treated tachyzoites. Thus, these bands and the corresponding regions in the tachyzoite extract blots were cut out and subjected to LC-MS and database mining for protein identification. Table 5 shows the results of this immunoproteomics approach for the 90 and 38 kDa band. Obviously, several proteins were identified. However, two filters were applied, namely (i) the approximate reported molecular weight, and (ii) for each candidate antigen the ratio of abundance in MNC vs. tachyzoite extract (MNC/T) of 2 or above as determined for each antigen. The resulting proteins that are overexpressed in MNCs are shown in Table 5. Overall, this indicates that the antibody response of live-vaccinated and BKI-1294 treated mice differed from the response of mice that were only live-vaccinated, with specific proteins recognized in MNCs but not in tachyzoite extracts.

**Table 5:** List of proteins found to be potentially present in bands specifically immunoreactive in MNC extracts, but missing in tachyzoite extracts, using antisera of live-vaccinated and BKI-1294 treated mice.

**Band**	**MNC/T**	**Accession No**.	**Protein name**	**Putative MW (kDa)**	**Score (AA)**
**(kDa)**					
90 ± 10	2.8	NCLIV_049900	Myosin C	93	107
	29.7	NCLIV_069130	Uncharacterized protein	89	11
	6.1	NCLIV_030050	Uncharacterized protein	97	55
	29.7	NCLIV_007010	Cathepsin C2, putative TgCPC2	83	38
	9	NCLIV_014430	Uncharacterized protein	80	22
38 ± 4	2.5	NCLIV_056430	Solute carrier family 25, member 10, related	39	86
	70.3	NCLIV_054830	Uncharacterized protein	35	114
	61.2	NCLIV_066970	Putative enoyl acyl carrier reductase	42	61

*Results show only those proteins that have an approximate putative molecular weight corresponding to the respective band, and are more than 2 times higher expressed in MNC compared to tachyzoites*.

## Discussion

In this study, we investigated the long-term effects of BKI-1294 treatment in BALB/c mice live-vaccinated with Nc-Liv tachyzoites with regard to safety and efficacy against re-infection with Nc-Sp7 tachyzoites during pregnancy. The 5 days treatment was done according to previous publications, in which the drug was applied 2 days following primary infection, showing highly promising efficacy in pregnant mice (19, 20). Our goal was to reproduce these findings and then study the long-term effects upon re-infection. While earlier studies have focused on the efficacy of BKI-treatments in animals that were infected during pregnancy, this is the first investigation on how BKI-1294 affects live-vaccinated mice. As anticipated from previous studies (20), BKI-1294 treatment applied shortly after live-vaccination reduced the cerebral parasite load in the dams, but had an unexpected detrimental impact on safety and efficacy against challenge infection during pregnancy, resulting in neonatal pup mortality and vertical transmission despite significantly decreased cerebral parasite load. IFN-y, IL-10, and IL-12-levels were consistently lower in live-vaccinated and BKI-1294 treated animals compared to non-treated mice. In addition, live vaccinated and BKI-1294 treated mice exhibited an antibody recognition pattern in MNC extracts that was different from non-drug-treated mice, and two protein bands in MNC extracts that were only recognized by sera of live-vaccinated mice treated with BKI-1294 were analyzed by proteomics.

*Neospora* live-vaccines are regarded as the most effective means of preventing neosporosis in mice as well as in cattle (1, 5, 38). Experimentally assessed live-vaccines include mostly naturally attenuated strains of low-virulence (4, 6–8, 39), strains that have been cultured during longer time periods and/or were applied at a subclinical dose (40, 41), gamma-irradiated mutants (42), a temperature sensitive strain induced by chemical mutagenesis (43, 44), and also a genetically modified strain (45). In this study, live-vaccination was carried out with Nc-Liv, known to be a highly virulent strain, that is reported to exhibit pup mortality and vertical transmission rates of 100% when inoculated into pregnant mice at dose of 2 × 10^6^ tachyzoites (37). However, when inoculated at the lower dose of 10^5^ tachyzoites (see group D), Nc-Liv did not induce any pathology in the dams, did not affect fertility, did not induce either neonatal nor postnatal mortality in offspring mice, and no recrudescence or vertical transmission could be detected. However, when Nc-Liv live-vaccinated mice were treated with BKI-1294 (group C), neonatal mortality was increased to 12.5% and vertical transmission was detected in 6 out of 56 pups. Although these numbers are not statistically significant, they demonstrate that BKI-1294 treatment could either result in increased recrudescence, or the treatment after live-vaccination resulted in compromised immunity, allowing the parasites to persist at low numbers and continue proliferation during pregnancy.

In addition, while live-vaccination induced substantial protective immunity against infection during pregnancy, this protective effect was partially lost in the live-vaccinated and BKI-1294 treated group A, especially with regard to vertical transmission, with a statistically significant increase to over 21%. Whether these congenitally infected pups were infected with Nc-Liv (used for live-vaccination), with NcSpain-7 (used for re-infection during pregnancy), or both, was not elucidated. This could be done by microsatellite characterization of PCR-positive samples. However, since vertical transmission was also seen without re-infection during pregnancy, it is conceivable that some of these animals were infected with both strains.

Surprisingly, pup mortality and vertical transmission rates did not correlate with the cerebral parasite burden in the dams analyzed at the endpoint of this experiment. Group B, live-vaccinated and treated with BKI-1294, exhibited the lowest cerebral parasite load, confirming earlier results on *in vivo* efficacy of BKI-1294 (20). Cerebral parasite loads in groups C and D (vaccinated/re-infected and vaccinated/non-re-infected, respectively, but both without BKI-1294 treatments), were significantly higher. Nevertheless, these two groups exhibited zero postnatal mortality and no vertical transmission. Analyses of the antibody responses confirmed the impact of BKI-1294 treatments on parasite load. Measurements of IgG1 and IgG2a on day 14 post-live-vaccination showed decreased IgG1 levels in BKI-treated mice compared to non-treated mice, while IgG2a levels remained the same. IgG2a levels were also not significantly different between the treatment groups on day 16 of pregnancy and on day 30 post-partum but significant differences between some of the groups were detected with respect to IgG1 levels. A comparison of the IgG1/IgG2a ratios at day 16 of pregnancy revealed that those groups that were live-vaccinated and re-infected (groups A and C) exhibited a predominantly IgG1-biased response, which was much less pronounced in group A (treated with BKI-1294 following live vaccination). In contrast, those groups that were only live-vaccinated without re-infection (B and D), exhibited IgG2a-biased responses, which is indicative for a Th1 cellular immune response. This response was slightly less pronounced in the BKI-treated group B.

The impact of BKI-1294 on the cellular immune response was first investigated by analyzing, *in vitro*, the direct effects of BKI-1294 on the proliferative capacities of mitogen-activated B and T cells stimulated with LPS and ConA, respectively. The drug did not affect these cells directly. The cellular immune response against *N. caninum* infection involves first the activation of antigen presenting cells (APCs), especially macrophages and dendritic cells (DCs), and the expression of IFN-γ. IFN-γ then triggers the production of high amounts of pro-inflammatory mediators, which in turn mediate tachyzoite-to-bradyzoite differentiation (46, 47). By analyzing recall responses in medium supernatant of splenocytes of BKI-1294 treated and non-treated mice collected on day 26 p.i. and stimulated with *N. caninum* antigen extract, IFN-γ was indeed identified as the by far most abundant cytokine. In sera of live-vaccinated mice, treated or not with BKI-1294, and taken on days 4, 11 and 26 post-live vaccination, cytokine levels were very low on day 4, but IFN-γ and the pro-inflammatory cytokine IL-12 were the most abundant cytokines detected on day 11 p.i.. On day 26 p.i., IL-12 and the regulatory cytokine IL-10 were present at high levels in sera of non-treated mice, but at lower levels in BKI-1294 treated mice. IFN-γ on the other hand was almost undetectable at the later timepoint in both treated and non-treated mice. Thus, BKI-1294 treatment affected the Th1-biased cellular immune response in live-vaccinated mice, probably due to its inhibitory effects on the parasites, and this correlates well with the reduced cerebral parasite burden as observed in group B (live-vaccinated and treated) compared to group D (only live-vaccinated).

Following infection in an immunocompetent host, it is expected that *N. caninum* tachyzoites respond to immunological and physiological stress by differentiating into tissue cyst-forming bradyzoites, which undergo limited proliferation and remain viable for extended periods of time without causing inflammatory responses (1). Recrudescence, meaning bradyzoite-to-tachyzoite re-differentiation can then be caused by (partial) loss of immunocompetence such as during pregnancy, during which a shift from a Th1- to a Th2-biased immune response takes place (48–50). However, tissue cysts and recrudescence in chronically infected BALB/c mice has not been detected frequently. Previous studies demonstrated that BALB/c mice chronically infected with two isolates of different virulence transmitted the infection to their progeny only at a very low rate [8 and 17% of total pups, for the higher (Nc-Sp7) and lower virulence (NcSpainH-1) isolate, respectively] (51, 52). Thus, while *N. caninum* tissue cyst formation has been generally linked to an inflammatory, Th1-biased cellular immune response (47, 50), it is not clear whether these live-vaccinated mice actually harbored tissue cysts. Overall, there are few reports on tissue cyst formation in inbred mouse strains (53). The most successful protocols designed to produce *N. caninum* tissue cysts in mice involved immunosuppressed outbred mice (e.g., ICR mice) infected with Nc-Liv, which is known to form tissue cysts more efficiently than other isolates (54–56).

It is possible that no tissue cysts were formed in BKI-1294 treated mice. *In vitro*, BKI-1294 induces the formation of MNCs, which contain newly formed zoites that undergo continuous DNA replication (21). A likely scenario is that MNCs were formed also *in vivo*, and this could be responsible for the observed reactivation of the live-vaccine and the increased vertical transmission upon re-infection. Shotgun proteomics had shown that MNCs exhibited an altered proteome when compared to tachyzoites, and this drug-induced stage was termed baryzoite (22). Following *in vitro* treatment with BKI-1294 during 5 days, it took approximately 10 days of culture without drug for MNCs to disappear and tachyzoites with SAG1 surface expression to re-emerge (21). If similar MNCs were also formed *in vivo*, it is conceivable that this could interfere in the process of cerebral tissue cyst formation, and thus reactivation of the infection during pregnancy could be taking place in a different manner than in non-treated mice. However, further *in vivo* studies and more detailed molecular and (immuno-)histopathological analyses of different organs will be necessary to clarify this point.

One indication that MNC formation did take place is provided by the immunoproteomics approach applied in this study. Tachyzoite and MNC extracts were separated by SDS-PAGE and Western blots were incubated with sera from live-vaccinated and non-treated or BKI-1294 treated mice. Alignment of Western blots resulted in the identification of 2 bands in MNC extracts of ~90 and 38 kDa recognized only by sera of BKI-1294 treated mice. LC-MS and data base mining showed that these bands were comprised of several proteins with potentially upregulated expression in MNCs. However, recent comparative proteomics of *N. caninum* tachyzoites and MNCs did not pick up any of these proteins as differentially expressed in the two stages (22), indicating that their expression levels might be below the resolution limit of the shotgun proteomics approach. In any case, these proteins represent interesting candidates for the further characterization of MNCs and for studies on how this drug-induced MNC stage could interact with the host.

In conclusion, the treatment of live-vaccinated mice with BKI-1294 affected the cellular and humoral immune responses against infection. While the treatment with this compound reduced the cerebral parasite burden in live-vaccinated animals, the safety of the live vaccine was diminished, and decreased protection against re-infection during pregnancy was observed, while live-vaccination without drug treatment resulted in excellent safety and efficacy. The formation of drug-induced MNCs could be implicated in this process. Whether similar effects can be seen with other compounds is not yet clear and remains to be investigated. However, MNCs similar to those seen in this study were reported upon *in vitro* treatment of *T. gondii* tachyzoite-infected cells with diclazuril (57). Diclazuril is a triazonine compound that affects mitochondrial enzymes of the respiratory chain and other enzymes such as dihydrofolate reductase, which is involved in nucleotide biosynthesis (58). Potentially, the findings presented here could have implications for the treatment with BKI-1294, and potentially other drugs, of *N. caninum* infected animals that acquire re-infection during pregnancy, and follow-up studies should be done in a more relevant ruminant animal model.

## Data Availability Statement

All datasets generated for this study are included in the article/Supplementary Material.

## Ethics Statement

The animal study was reviewed and approved by Animal Welfare Committee of the Canton of Bern, Kantonales Veterinäramt, Münsterplatz 3a, Postfach. 3000 Bern 8.

## Author Contributions

AH and PW conceived and designed the study. AH coordinated the biological assays. PW, DI, NA, and AA-M carried out *in vitro* and *in vivo* experimental work. L-MO-M, WV, and KO provided the compound. JM, AA-M, and PW carried out statistical analysis. WV, KO, L-MO-M, AH, and PW did interpretation of results. AH and PW wrote the manuscript. All authors corrected and approved the manuscript.

## Conflict of Interest

WV is an officer of ParaTheraTech, Inc, a company that is striving to develop BKIs into animal therapeutics. The remaining authors declare that the research was conducted in the absence of any commercial or financial relationships that could be construed as a potential conflict of interest.
