# Supplementary figures and images for: The Impact of BKI-1294 Therapy in Mice Infected With the Apicomplexan Parasite Neospora caninum and Re-infected During Pregnancy
Source: Front Vet Sci. 2020 Oct 15;7:587570. doi: 10.3389/fvets.2020.587570 (PMC7593410; doi:10.3389/fvets.2020.587570)

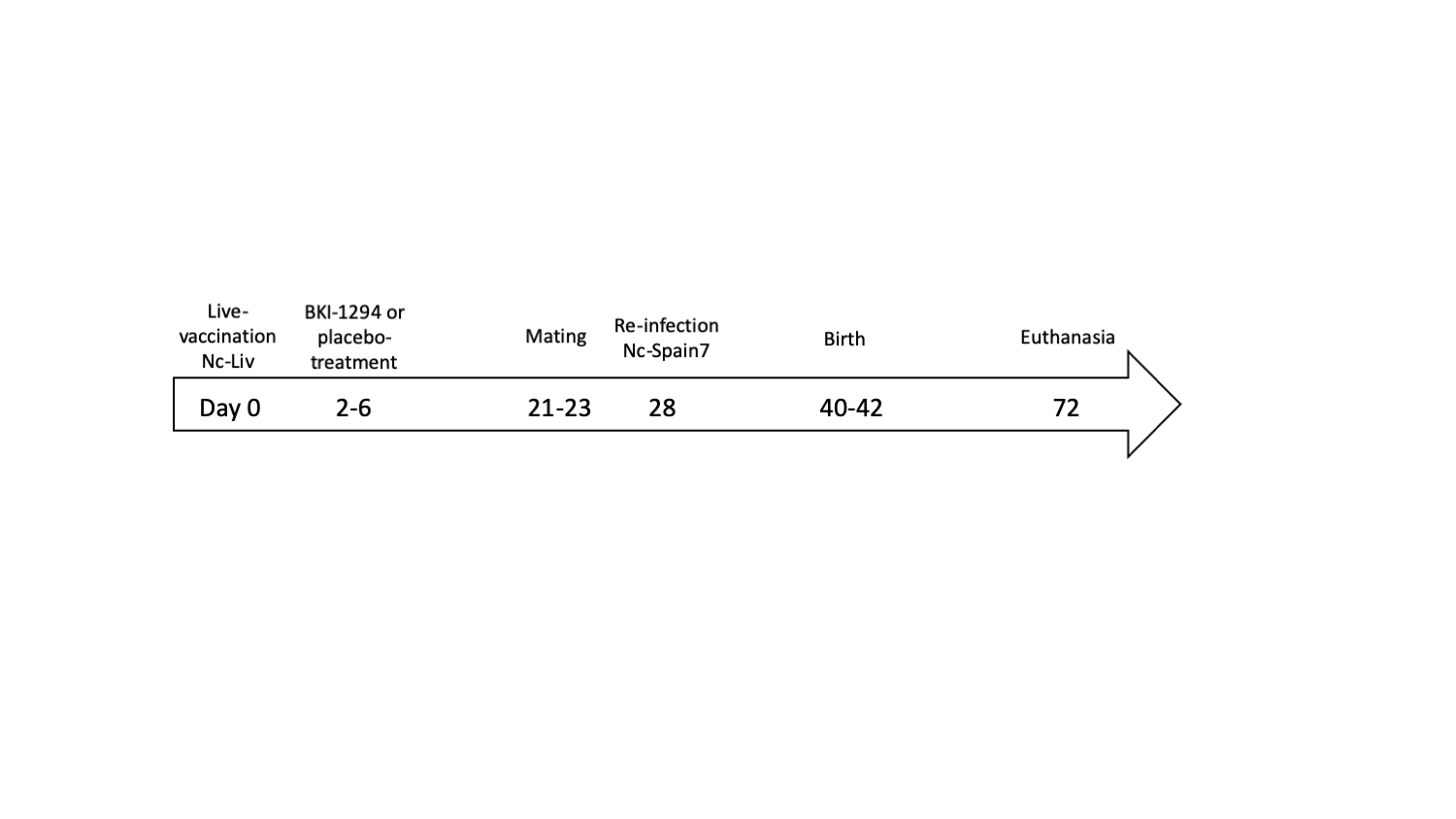

Supplement: Supplementary file 1 [file Image_1.TIFF]
